# Efficient Decellularization of the Full-Thickness Rat-Derived Abdominal Wall to Produce Acellular Biologic Scaffolds for Tissue Reconstruction: Promising Evidence Acquired from In Vitro Results

**DOI:** 10.3390/bioengineering10080913

**Published:** 2023-08-01

**Authors:** George Skepastianos, Panagiotis Mallis, Epameinondas Kostopoulos, Efstathios Michalopoulos, Vasileios Skepastianos, Chrysoula Palazi, Lucia Pannuto, Gerasimos Tsourouflis

**Affiliations:** 1 Plastic Surgery Department, EANP Metaxa, National Hospital of Athens, 51 Botatsi Street, 185 37 Pireus, Greece; skep-19@hotmail.com (G.S.); epamkostop67@hotmail.com (E.K.); bskepastianos@hotmail.com (V.S.); xrisa.pal@gmail.com (C.P.); 2 Center of Experimental Surgery, Biomedical Research Foundation Academy of Athens, 4 Soranou Ephessiou Street, 115 27 Athens, Greece; 3 Hellenic Cord Blood Bank, Biomedical Research Foundation Academy of Athens, 4 Soranou Ephessiou Street, 115 27 Athens, Greece; smichal@bioacademy.gr; 4 Queen Victoria Hospital NHS Foundation Trust, East Grinstead RH19 3DZ, UK; lucia.pannuto@gmail.com; 5 Second Department of Propedeutic Surgery, Medical School, University of Athens, 115 27 Athens, Greece; gerasimos.ts@gmail.com

**Keywords:** abdominal wall, decellularization, hernia, tissue reconstruction, biomechanical analysis, full-thickness abdominal wall scaffold

## Abstract

Background: Functional restoration of abdominal wall defects represents one of the fundamental challenges of reconstructive surgery. Synthetic grafts or crosslinked animal-derived biological grafts are characterized by significant adverse reactions, which are mostly observed after their implantation. The aim of this study was to evaluate the efficacy of the decellularization protocol to produce a completely acellular full-thickness abdominal wall scaffold. Methods: Full-thickness abdominal wall samples were harvested from Wistar rats and submitted to a three-cycle decellularization process. Histological, biochemical, and DNA quantification analyses were applied to evaluate the effect of the decellularization protocol. Mechanical testing and immunogenicity assessment were also performed. Results: Histological, biochemical, and DNA analysis results showed efficient decellularization of the abdominal wall samples after the third cycle. Decellularized abdominal wall scaffolds were characterized by good biochemical and mechanical properties. Conclusion: The data presented herein confirm the effective production of a rat-derived full-thickness abdominal wall scaffold. Expanding this approach will allow the exploitation of the capacity of the proposed decellularization protocol in producing acellular abdominal wall scaffolds from larger animal models or human cadaveric donors. In this way, the utility of biological scaffolds with preserved in vivo remodeling properties may be one step closer to its application in clinical studies.

## 1. Introduction

The restoration of abdominal wall defects as a result of trauma, infection, congenital conditions, complications of abdominal surgeries, neoplastic diseases, and others belongs among the most routinely performed surgeries in the general population [1]. Among these defects, incisional hernias that occur after laparotomy are exhibited in 5–20% of patients, and their repair represents a highly demanding reconstructive surgery [2]. It is estimated that more than 700,000 surgeries for abdominal wall reconstruction are performed in the United States annually and more than 20 million are performed globally each year [1,2,3,4].

Mostly, abdominal wall surgery and herniation can lead to significant complications, including muscle weakness and collagen ratio disturbance [5]. Regarding the latter, it has been already described in the literature that the amount of collagen I reduces, while the amount of collagen III increases, resulting in alterations in abdominal wall tensile strength and mechanical stability [5,6,7,8]. In accordance with the aforementioned information, the proper reconstruction of abdominal wall defects requires the use of scaffolds characterized by specific biomechanical properties to support tissue regeneration and deep wound healing [9]. Nowadays, a great number of commercially produced synthetic scaffolds are used as implants for abdominal wall reconstruction [1,10,11]. Synthetic scaffolds can be produced using either biodegradable or non-biodegradable materials. Polyglycolic acid (PLGA) and polyglactin 910 (PLGA 910) are biodegradable materials, which are degraded through the hydrolysis process [12,13,14]. In this way, tissue-resident macrophages can efficiently degrade these materials into monomers, which further can be deposited into the regenerated ECM. However, abdominal wall reconstruction performed with biodegradable materials results mostly in scar tissue formation at the injury site [15,16,17]. This in turn can result in complications due to scar tissue formation, impairing tissue integrity and mechanical resistance. Unfortunately, new reconstructive surgery to replace the formed scar tissue is required [18]. In contrast, polypropylene (PP) and expanded tetrapolyfluorethylene (ePTFE) belong to the category of non-biodegradable materials with good biomechanical properties and tissue reinforcement [19,20,21]. Although these materials have good properties, severe adverse reactions, such as calcification and host immune system activation, have been reported in the majority of cases [1]. Other factors, such as the body mass index (BMI) and post-operative wound infection, also should be considered for the selection of a proper scaffold for the functional reconstruction of the abdominal wall [22]. Indeed, patients with BMI > 30, accompanied by other health issues, such as diabetes mellitus and chronic obstructive pulmonary disease (COPD), are characterized by a higher risk ratio of ventral hernia formation due to the increased stress of the abdominal wall [22]. In addition, patients with a smoking habit, prior ventral hernia restorations, and infections related to hernia repair may exhibit a great number of postoperative complications following the use of either biodegradable or non-biodegradable materials. Moreover, a meta-analysis study conducted by Darahzereski et al. [23] showed that synthetic materials used for hernia restoration have an infection rate of 36.5% compared to 10.9% of biological scaffolds. 

Considering the aforementioned findings, scaffolds derived from different origins, e.g., a natural plant-based or an animal-derived ECM, must be evaluated as a potential new strategy for abdominal wall reconstruction. In the context of tissue regeneration, biological scaffolds composed of a native or glutaraldehyde crosslinked ECM have been used in the past with limited success [24,25]. However, after long-term implantation, aberrant host immune responses have also been reported [26,27]. Immune system activation includes mostly the polarization of macrophages into the M1 phenotype, the activation of CD4 T cells, and the migration of Th1 and B cells to the implantation site, which further leads to calcification and tissue rejection [26,27,28]. However, the production of biological scaffolds using advanced tissue engineering approaches, such as decellularization, may overcome the aforementioned issues [29]. Decellularization relies on the use of a combination of physical and chemical methods, leading to cellular loss and the production of an acellular matrix [30]. The decellularized ECM can beneficially support cell adhesion, survival, proliferation, and differentiation compared to synthetic or crosslinked scaffolds [31]. The former is related also to better biomechanical properties, which can lead to improved tissue reconstruction at the injury site [32,33]. Nowadays, the use of scaffolds obtained from decellularized matrices is comprehensively investigated as a pioneer tissue engineering approach and alternative therapeutic strategy for several life-threatening diseases, such as cardiovascular disease (CVD), bone defects, and dental and craniofacial issues [29,30,31,32,33,34,35]. Different research groups have elaborated on protocols for the successful decellularization of abdominal wall scaffolds of animal origin [36,37,38,39]. The application of a decellularization protocol can be easily performed in a great variety of tissues, although the preservation of the full-thickness ECM comprises a highly demanding task. For this reason, the literature regarding the successful production of full-thickness abdominal wall scaffolds is limited [36,37,38,39], yet the needs for reconstructive surgery demand the production of such structures. In the past, perfusion decellularization was proposed for the production of whole-organ scaffolds; however, this process is laborious and requires advanced equipment and well-trained personnel [40]. Despite the broad use of the decellularization method in different tissues and organs, serious issues concerning extensive ECM damage in the produced scaffolds have still been reported [39,40,41,42]. Thus, a damaged ECM is characterized by advanced difficulties in (a) performing functional restoration of the injury site, (b) repopulation by resident cells, and (c) achieving in vivo remodeling based on the new microenvironment requirements. Eventually, this could lead to unfavorable results related to the implanted scaffold, such as extensive calcification, an immune response, and graft rejection [39,40,41,42]. Based on our previous experience, we validated a cost-effective perfusion-free decellularization method (avoiding extended incubation times of the ECM with the decellularization solutions), which can potentially be applied as an alternative method for the production of full-thickness abdominal wall scaffolds [40].

The aim of this study was to test the efficacy of the production of an acellular scaffold originating from full-thickness rat-derived abdominal wall samples. For this purpose, our optimized decellularization protocol consisting of three cycles was applied to full-thickness rat-derived abdominal wall samples. The evaluation of the decellularized abdominal wall ECM was performed using methods such as histological analysis and biochemical and DNA quantification. Furthermore, the biomechanical properties of the abdominal wall before and after the decellularization approach were also investigated. The obtained results may lead to significant conclusions regarding full-thickness abdominal wall decellularization to be used in large-scale experimental procedures. 

## 2. Methods

### 2.1. Abdominal Wall Excision

Full-thickness abdominal wall samples (including all muscle layers) were harvested from Wistar rats (*n* = 30), weighing 300–400 g, under aseptic conditions. All animals were provided by the Animal Facility of the Biomedical Research Foundation Academy of Athens (BRFAA). Well-trained personnel of the animal facility handled and cared for the animals according to the international guidelines of animal care and conformed with the Declaration of Helsinki. In addition, this study was approved by the Bioethics Committee of the BRFAA (ref. 02-2021). Briefly, the animals were euthanized, and using sterile instruments, a paramedian incision in the anterior abdominal wall was performed in order to expose the external oblique muscles. The abdominal wall samples were extracted in dimensions of 4 × 5 cm and then stored in phosphate-buffered saline (PBS; Sigma-Aldrich, Darmstadt, Germany) 1× supplemented with 1% penicillin–streptomycin (P-S; Sigma-Aldrich, Darmstadt, Germany) until further processing.

### 2.2. Decellularization of Whole Rat Abdominal Wall Samples

Whole abdominal wall samples (*n* = 30) were decellularized using an already published protocol validated previously in our lab [40]. Whole rat abdominal wall samples were incubated with decellularization buffers under vigorous agitation at 150 rpm. The decellularization procedure involved the incubation of the samples in CHAPS buffer (pH 7; 8 mM CHAPS, 1 M NaCl, and 25 mM EDTA in PBS 1× (Sigma-Aldrich, Darmstadt, Germany) for 18 h at room temperature (RT). Next, the rat abdominal wall samples were washed 3 times (10 min each time) with PBS 1× (Sigma-Aldrich, Darmstadt, Germany) under continuous agitation (150 rpm) at RT. The samples were incubated in SDS buffer (pH 7; 1.8 mM SDS, 1 M NaCl, and 25 mM EDTA in PBS 1×; Sigma-Aldrich, Darmstadt, Germany) for another 18h at RT. The samples were again washed 3 times (10 min each time) with PBS 1× (Sigma-Aldrich, Darmstadt, Germany) under continuous agitation (150 rpm) at RT. Finally, the samples were placed in a solution containing a-Minimum Essentials Medium (a-MEM; Sigma-Aldrich, Darmstadt, Germany) supplemented with 40% *v*/*v* fetal bovine serum (FBS; Gibco, Thermo Fisher Scientific, Waltham, MA, USA) for 36 h at 37 °C and washed again, as described before. The whole decellularization procedure was repeated another 2 times (in total, 3 decellularization cycles, *n* = 5 samples/decellularization cycle).

### 2.3. Histological Analysis

Non-decellularized (*n* = 10) and decellularized (*n* = 15) whole rat abdominal wall samples (obtained from the 1st, 2nd, and 3rd decellularization cycles) were fixed overnight using 10% *v*/*v* neutral formalin buffer (Sigma-Aldrich, Darmstadt, Germany), followed by washes with distilled water. After this step, all samples were dehydrated in an increasing series of alcohol buffers, paraffin-embedded, and finally sectioned at 5 μm. The production of acellular abdominal wall scaffolds was evaluated based on the performance of specific histological stains. For this purpose, hematoxylin and eosin (H&E; VWR, Avantor, Radnor, PA, USA), Sirius Red (SR; VWR, Avantor, Radnor, PA, USA), Orcein (OS; VWR, Avantor, Radnor, PA, USA), and Toluidine Blue (TB; VWR, Avantor, Radnor, PA, USA) were used for the evaluation of the abdominal wall ECM, collagen fibers, elastin fibers, and sulfated glycosaminoglycans (sGAGs), respectively. The sections of non-decellularized and decellularized abdominal wall samples were deparaffinized, rehydrated, stained with each stain, and mounted with a cover slide. Whole rat abdominal wall samples were characterized by a combination of longitudinally and circumferentially oriented fibers, and for this purpose, representative images were obtained. Images were acquired using the Leica DM L2 light microscope (Leica Microsystems, Weltzar, Germany) and processed with Image J v.1.46 (Wane Rasband, National Institutes of Health, Bethesda, ML, USA).

To further evaluate the impact of each decellularization cycle on collagen type I, indirect immunofluorescence was performed. Briefly, the sections of all study samples were deparaffinized, rehydrated, and blocked. Next, incubation with the primary monoclonal antibody against collagen type I (1:1000; Sigma-Aldrich, Darmstadt, Germany) was performed. Brief washes were performed, and then incubation with the FITC-conjugated IgG secondary antibody (1:500; Sigma-Aldrich, Darmstadt, Germany) was performed. Finally, cell nuclei were stained with DAPI (Sigma-Aldrich, Darmstadt, Germany), and then, the samples were dehydrated and glycerin-mounted. The samples were examined under the LEICA SP5 II confocal microscope, and representative images were acquired with LAS Suite v2 software (Leica Microsystems, Weltzar, Germany).

### 2.4. Biochemical Analysis

The biochemical analysis included the evaluation of the collagen (hydroxyproline) and sGAG content. Moreover, representative areas of non-decellularized and decellularized whole rat abdominal wall samples (1 × 1 cm) were selected from each sample. Specifically, for collagen content quantification, non-decellularized (*n* = 10) and decellularized (*n* = 30, *n* = 10/decellularization cycle) samples were digested in papain solution (125 μg/mL; Sigma-Aldrich, Darmstadt, Germany) at 60 °C for 10 min or until full digestion of the samples. The collagen content was estimated based on the hydroxyproline content determined with the hydroxyproline assay kit (MAK008, Sigma-Aldrich, Darmstadt, Germany) following the manufacturer’s instructions. The sGAG content of non-decellularized (*n* = 10) and decellularized (*n* = 30, *n* = 10/decellularization cycle) samples was determined, as previously described. Briefly, the samples were digested with 1 mL of lysis buffer (0.1 M Tris pH 8, 0.2 M NaCl, and 5 mM EDTA in PBS 1× (Sigma Aldrich, Darmstadt, Germany)) and 25 mg/mL of proteinase K (Sigma-Aldrich, Darmstadt, Germany) at 56 °C for 12 h. Inactivation of proteinase K was performed at 95 °C for 5 min. The 1% *w*/*v* dimethylene blue stain (Sigma-Aldrich, Darmstadt, Germany) was used, and the determination of the sGAG content of each sample was performed photometrically at 525 nm. Finally, the sGAG concentration of each sample was estimated through interpolation to a standard curve based on chondroitin sulfate standards of 3, 6, 12, 25, 50, 100, and 150 μg/mL.

### 2.5. DNA Quantification

For DNA quantification, non-decellularized (*n* = 10) and decellularized (*n* = 30, *n* = 10/decellularization cycle) samples were initially digested using proteinase K (25 μg/mL; Sigma-Aldrich, Darmstadt, Germany) in PBS 1× (Sigma-Aldrich, Darmstadt, Germany) at 56 °C for 12 h, followed by inactivation at 96 °C for 5 min. Next, isolation of the DNA of each sample was performed, and the DNA was finally eluted in 50 μL RNAse-free water (Sigma-Aldrich, Darmstadt, Germany). The DNA content of each sample was spectrophotometrically determined using Nanodrop (Thermo Fischer Scientific, Waltham, MA, USA) at 260/280 nm. Also, the DNA quantification results were verified using DNA electrophoresis on 1% *w*/*v* agarose gel. Images were acquired with the UVITEC Imaging System (Cleaver Scientific, Warwickshire, UK).

### 2.6. Biomechanical Analysis

The biomechanical analysis of non-decellularized (*n* = 5) and decellularized (*n* = 5/cycle) whole rat abdominal wall samples was performed to evaluate their mechanical properties. The biomechanical analysis involved the uniaxial testing of the samples, which was performed in a Zwick/Roell tensile tester (model Z 0.5, Zwick GmbH & Co. KG, Ulm, Germany) equipped with a 100 N load cell. Non-decellularized and decellularized samples were cut into longitudinal strips (l = 40 mm, w = 10 mm) and placed before the analysis in prewarmed Krebs–Ringer solution (Gibco, Thermo Fisher Scientific, Waltham, MA, USA) at 37 °C. During the biomechanical testing, the strips were continuously sprayed with PBS 1× (Sigma-Aldrich, Darmstadt, Germany). The strips derived from non-decellularized and decellularized samples were clamped at their ends with sandpaper to avoid sample slippage, under zero strain on the mechanical device, which produced a preloading value of 0.005 N, before data collection. All samples were preconditioned for 10 cycles at a rate of 10 mm/min. Sample extension (Δl, in mm) accompanying the generated load (F, in Newtons) was converted to engineering strain (ε) and engineering stress (σ in MPa). Elastin (El-E) and collagen (Col-E) phase slopes, transition stress (σΤrans) and strain (εTrans), ultimate tensile strength (σUTS), and failure strain (εUTS) were used to analyze the stress–strain behaviour of the studied samples.

### 2.7. Determination of Acellular Whole Abdominal Wall Immunogenicity

To determine the immunogenicity of decellularized whole abdominal wall scaffolds, the following assays were performed. Indirect immunofluorescence against α-gal (1:500; Sigma-Aldrich, Darmstadt, Germany) and collagen type I (1:1000; Sigma-Aldrich, Darmstadt, Germany) was performed, as previously described. Secondary PE-conjugated monoclonal antibody (1:50) against α-gal epitopes and FITC-conjugated monoclonal antibody (1:500) against collagen type I were used for the detection of the aforementioned proteins. Finally, all samples were dehydrated, glycerin-mounted, and examined using the LEICA SP5 II confocal microscope coupled with LAS Suite v2 software (Leica Microsystems, Weltzar, Germany). In addition, the detection of α-gal epitopes was performed using the ELISA method for additional verification of indirect immunofluorescence results. Initially, non-decellularized (*n* = 5) and decellularized (*n* = 5/each cycle) samples were fully lysed in 1% *w*/*v* papain medium (Sigma-Aldrich, Darmstadt, Germany) at 37 °C for a maximum of 10 h. Next, the lysates passed through 0.25 nm filters. After this step, the ELISA method was performed following the manufacturer’s instructions (ab239716, Abcam, Cambridge, UK). Furthermore, the gene expression profile in non-decellularized and decellularized samples was determined. Total mRNA was isolated from non-decellularized (*n* = 5) and decellularized (*n* = 5/each cycle) samples using TRI-reagent (Sigma-Aldrich, Darmstadt, Germany) according to the manufacturer’s instructions. The concentration of isolated mRNA was photometrically determined. Next, at least 400 ng of total mRNA was used as a template for cDNA production using the Omniscript reverse transcription kit (Qiagen, Hilden, Germany). PCR was performed using the Taq PCR master mix (cat. no. 201443, Qiagen, Hilden, Germany) on a Biometra T Gradient Thermoblock PCR Thermocycler (Biometra, Gottingen, Germany), with a final volume of 25 μL for each PCR. The PCR program involved the following steps: (1) denaturation at 95 °C for 15 min, (2) denaturation at 94 °C for 30 s, (3) annealing at 60–62 °C for 90 s, and (4) extension at 72 °C for 3 min. The number of cycles used in this program was 35. The primer pairs for each PCR are listed in Table 1. Finally, the PCR products were analyzed with electrophoresis on agarose gel (1% *w*/*v*; Sigma-Aldrich, Darmstadt, Germany). In addition, to evaluate the immunogenicity of non-decellularized and decellularized abdominal wall samples, flow cytometric analysis was performed. Initially, non-decellularized (*n* = 5) and decellularized (*n* = 5/each cycle) samples were fully digested in 1% *w*/*v* collagenase medium at 37 °C for a maximum of 4 h. The isolated cells were washed 3 times with PBS 1× (Sigma-Aldrich, Darmstadt, Germany) and then were submitted for flow cytometric analysis. Specifically, cells obtained from all samples were analyzed for the expression of RT1 class I, RT1 class II, CD31, and α-SMA. Monoclonal antibodies against RT1 class I and α-SMA were conjugated with fluorescein isothiocyanate (FITC), while those against RT1 class II and CD31 were conjugated with phycoerythrin (PE) and peridinin–chlorophyll–protein (PERCP), respectively. Anti-RT1 class I and class II were purchased from Biocompare (South San Francisco, CA, USA). The rest of the monoclonal antibodies were purchased from BD Biosciences (Franklin Lakes, NJ, USA) and analyzed at a minimum of 10,000 events in FACS Calibur (BD Biosciences, Franklin Lakes, NJ, USA).

### 2.8. Statistical Analysis

The statistical analysis in this study was performed using GraphPad Prism v 6.01 (GraphPad Software, San Diego, CA, USA). Comparisons regarding the hydroxyproline, sGAG, and DNA content and also comprehensive biomechanical analysis were performed using the Kruskal–Wallis test. Furthermore, validation of our results involved the use of the unpaired nonparametric Mann–Whitney U test. Statistically significant differences between group values were considered when the *p* value was less than 0.05. Indicated values were presented as the mean ± standard deviation.

## 3. Results

### 3.1. Histological and Biochemical Evaluation of Decellularized Full-Thickness Rat-Derived Abdominal Wall

**Figure 1:**
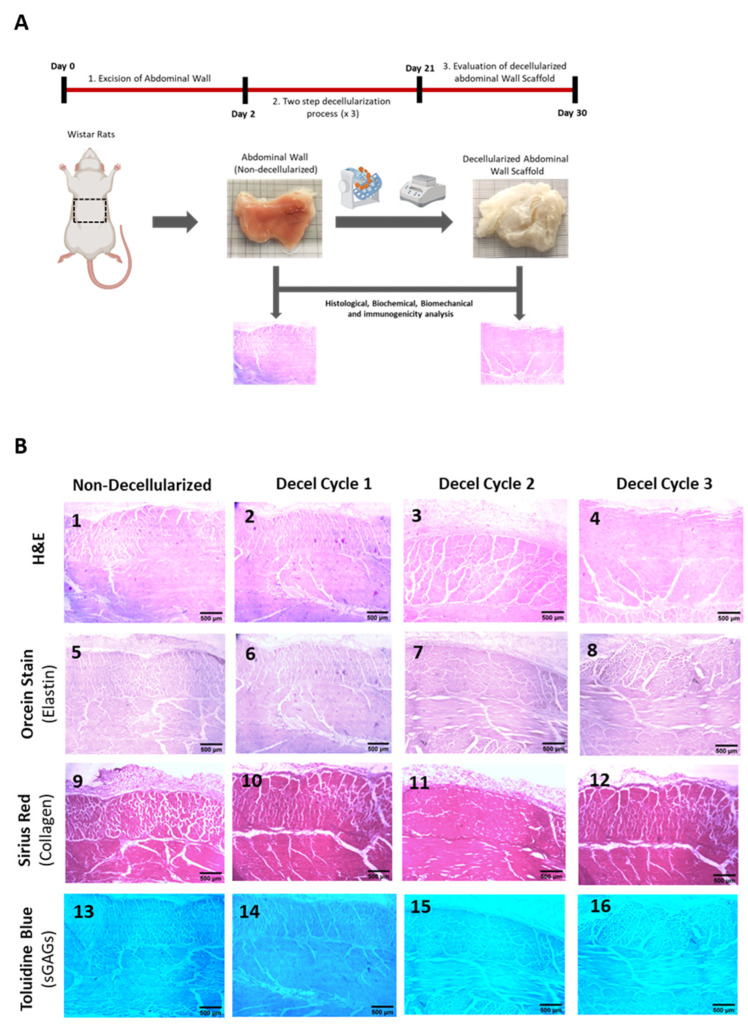
Assessment of decellularization of the full-thickness rat-derived abdominal wall. Overview of the applied decellularization protocol for producing acellular full-thickness abdominal wall scaffolds (**A**). Histological analysis involving the performance of H&E (**B1**–**B4**), Orcein (**B5**–**B8**), Sirius Red (**B9**–**B12**), and Tolouidine Blue (**B13**–**B16**) stains in non-decellularizaed and decellularized full-thickness abdominal wall samples. All images are presented with the original magnification of 2.5× and scale bars of 500 μm.

To properly evaluate the decellularization process of the full-thickness rat-derived abdominal wall, histological and biochemical analyses were initially performed. The abdominal wall represents a complex tissue including a combination of circumferential and longitudinal fibers, and for this purpose, the histological analysis involved the evaluation in both axes. A total of three decellularization cycles was required for the efficient production of acellular whole abdominal wall scaffolds. By the end of the third decellularization cycle, the acellular whole abdominal wall scaffolds appeared translucent, indicating furthermore the proper elimination of the resident cellular populations (Figure 1). 

**Figure 2:**
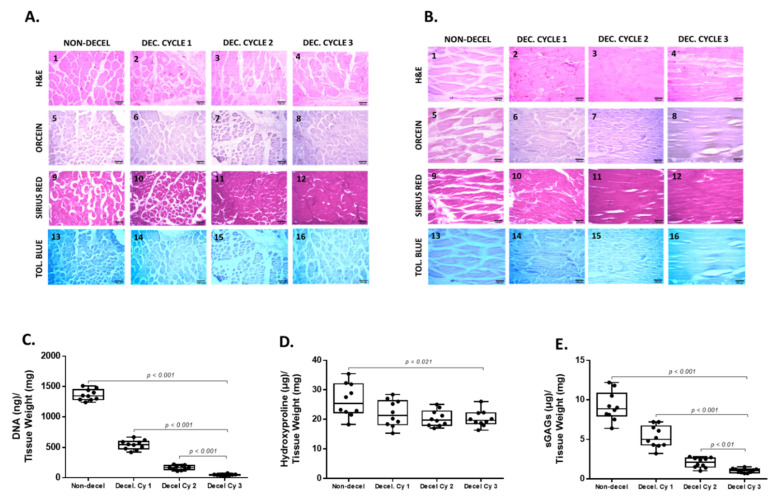
Evaluation of the decellularization approach for producing acellular whole rat abdominal wall scaffolds using histological and biochemical approaches. Representable images of non-decellularized and decellularized samples stained with H&E in circumferential (**A1**–**A4**) and longitudinal (**B1**–**B4**) axes. Hematoxylin staining was only evident in non-decellularized samples, which further confirmed the presence of cell nuclei. No hematoxylin staining was weaker after each decellularization cycle. Decellularized samples after the 3rd cycle were characterized by the absence of hematoxylin stain, thus confirming further the removal of cellular materials. No alteration in Orcein staining between non-decellularized and decellularized samples was observed either in the circumferential (**A5**–**A8**) or in the longitudinal (**B5**–**B8**) axis. Collagen fibers appeared to be retained both in composition and in alignment, as indicated by Sirius Red staining either in the circumferential (**A9**–**A12**) or in the longitudinal (**B9**–**B12**) axis. Representable images of non-decellularized and decellularized samples stained with Toluidine Blue, either in the circumferential (**A13**–**A16**) or in the longitudinal (**B13**–**B16**) axis. The staining intensity of Toluidine Blue became weaker after each decellularization cycle, compared to the non-decellularized samples, indicating the removal of sGAGs. All images were presented with the original magnification of 10× and scale bars of 100 μm. DNA quantification (**C**) and biochemical analysis, including the determination of the hydroxyproline content (**D**) and the sGAG content (**E**), were performed to validate further the decellularization process. Statistically significant differences regarding the DNA content (*p* <0.001), hydroxyproline content (*p* = 0.021), and sGAG content (*p* < 0.001) were observed between non-decellularized and decellularized samples.

In addition, the ECM integrity of the decellularized abdominal wall scaffolds was retained properly among all layers of the tissue (Figure 1 and Figure 2). No cracks or other destruction evidence was observed in the produced decellularized whole rat abdominal wall scaffolds (Figure 1 and Figure 2). To better assess the decellularization efficacy, H&E staining was initially performed. The removal of the resident cells was initially observed after the first decellularization cycle; however, total cell and nuclear material elimination was detected by the end of the third decellularization cycle (Figure 2). Once the cell elimination was confirmed with H&E staining, further evidence regarding the ECM composition of the acellular whole abdominal wall scaffolds was obtained based on the performance of Orcein, Sirius Red, and Toluidine Blue stains. Moreover, Sirius Red staining revealed the preservation of collagen fibers in both circumferential and longitudinal directions in the decellularized abdominal wall scaffolds. No destruction or change in the collagen fiber alignment was detected between native and decellularized abdominal wall scaffolds (after the third cycle). However, the abdominal wall is characterized by the presence of a low elastin amount, and in our case, it was difficult to be detected based on the performance of the Orcein stain; however, no significant change was observed between non-decellularized and decellularized abdominal wall samples (after the third cycle). In contrast, sGAGs in the decellularized abdominal wall appeared to be affected by each decellularization cycle (Figure 2). Indeed, after the accomplishment of each decellularization cycle, Toluidine Blue staining (specifically stains sGAGs) was weaker. Finally, the acellular full-thickness abdominal wall scaffolds produced (after the third cycle) were characterized by a significant loss of sGAGs, as indicated by the Toluidine Blue staining.

**Figure 3:**
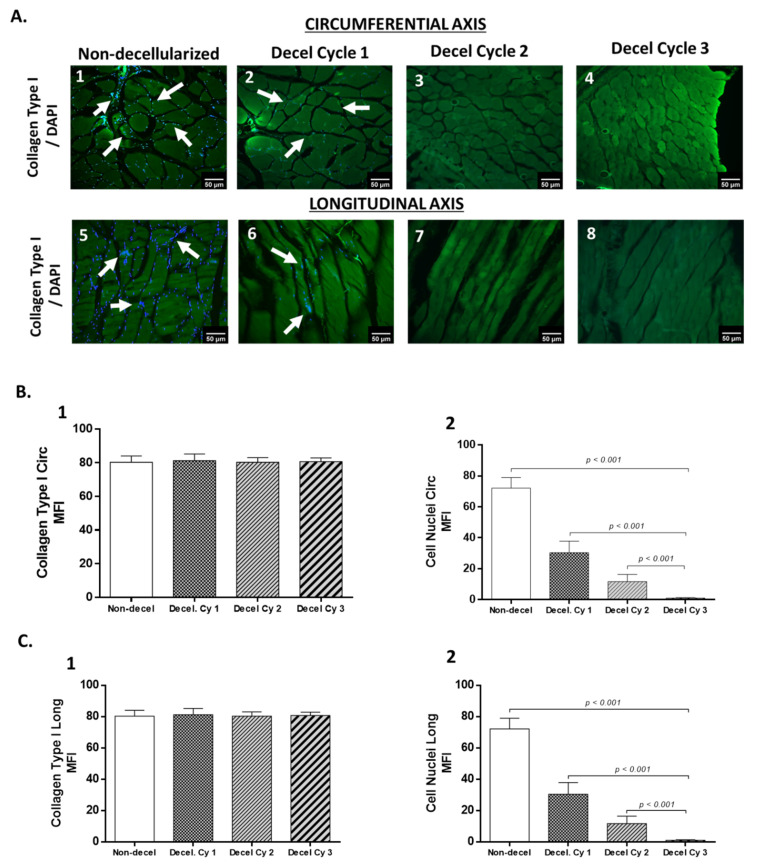
Evaluation of the decellularized whole abdominal wall scaffolds with indirect immunofluorescence. Indirect immunofluorescence against collagen type I (**green**), in combination with DAPI stain (**blue**), was applied both in non-decellularized and in decellularized whole abdominal wall scaffolds (after the 1st, 2nd, and 3rd cycles) in the circumferential and longitudinal axes (**A1**–**A8**). White arrows indicate the presence of cell nuclei. Cell nuclei were evident in non-decellularized and decellularized samples after the 1st cycle. No cell nuclei were detected in decellularized samples after the 2nd and 3rd decellularization cycles in both axes. The images are presented with the original magnification of 20× and scale bars of 50 μm. The MFI of detected collagen and DAPI stain of all samples in both circumferential and longitudinal axes was determined, as shown in graphs (**B1**,**B2**,**C1**,**C2**). Statistically significant differences were observed between non-decellularized and decellularized samples regarding the DAPI stain both in the circumferential (*p* < 0.001) and in the longitudinal (*p* < 0.001) axis.

DNA quantification and biochemical analysis were performed to validate further the composition of the decellularized whole rat abdominal wall. Specifically, the DNA content after each decellularization cycle was statistically significantly reduced (*p* < 0.001). Non-decellularized samples contained 1363 ± 87 ng of DNA/mg dry tissue weight (Figure 2 and Table S1), while the DNA content of decellularized samples was 542 ± 73, 167 ± 35, and 48 ± 12 ng DNA/mg dry tissue weight after the first, second, and third cycles, respectively. The hydroxyproline content, which corresponds to collagen, was reduced statistically significantly (*p =* 0.021) in decellularized samples. The hydroxyproline content of non-decellularized samples was 26 ± 5 μg/mg dry tissue weight. The hydroxyproline content of decellularized samples after the first, second, and third cycles was 22 ± 4, 20 ± 3, and 20 ± 2 μg hydroxyproline/mg dry tissue weight, respectively (Figure 2 and Table S1). The sGAG content of non-decellularized samples was 9.1 ± 1.7 μg sGAGs/mg dry tissue weight, while decellularized samples had 5.3 ± 1.7, 2.1 ± 0.6, and 1.1 ± 0.3 μg sGAGs/mg dry tissue weight, respectively (Figure 2 and Table S1). Statistically significant differences were observed in the sGAG content between non-decellularized and decellularized samples of the whole abdominal wall (*p* < 0.001). In addition, indirect immunofluorescence against collagen type I in combination with the DAPI stain was performed to validate further the decellularization process. The results of indirect immunofluorescence showed the preservation of collagen type I in decellularized whole abdominal wall scaffolds (after the first, second, and third cycles) in both axes. However, the DAPI stain, which was specific for the cell nuclei, gradually lost its intensity signal after the first decellularization cycle (Figure 3). To confirm these results, the determination of the MFI of collagen type I and DAPI stain was performed in all samples. In the circumferential axis, non-decellularized and decellularized samples after the first, second, and third cycles were characterized by 80.3 ± 3.5, 81.2 ± 3.8, 80.3 ± 2.6, and 80.7 ± 2.1 MFI for collagen type I and 72.1 ± 6.8, 30.4 ± 7.1, 11.7 ± 4.4, and 1.1 ± 0.3 for the DAPI stain, respectively (Figure 3). In the longitudinal axis, the MFI of non-decellularized and decellularized samples after the first, second, and third cycles was 81.4 ± 7.7, 80.6 ± 3.4, 80.7 ± 2.4, and 80.6 ± 2.3 for collagen type I and 73.7 ± 4.7, 27.3 ± 4.1, 9.7 ± 3.1, and 0.9 ± 0.3 for the DAPI stain, respectively (Figure 3). Statistically significant differences were observed between the study samples only for the DAPI stain in both axes (*p* < 0.001).

### 3.2. Evaluation of Biomechanical Properties

**Figure 4:**
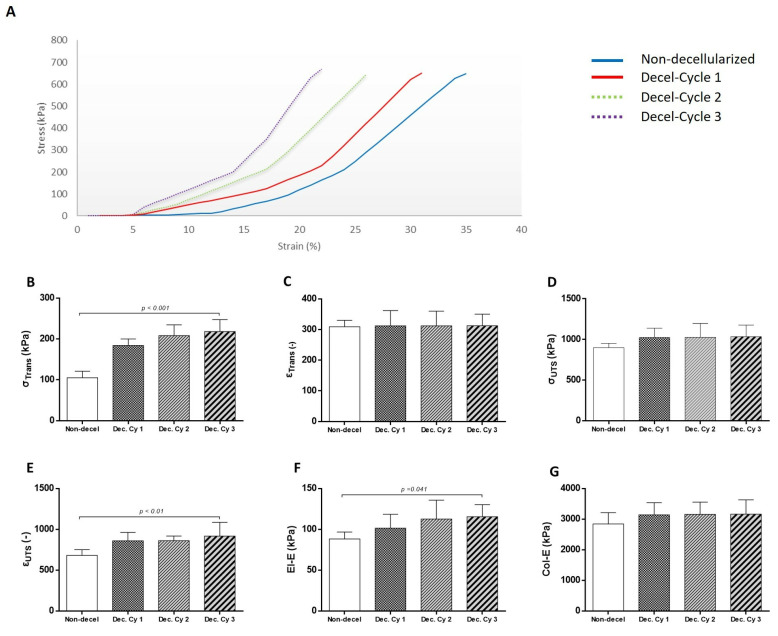
Biomechanical evaluation of non-decellularized and decellularized whole abdominal wall scaffolds. Representative stress–strain curves of non-decellularized and decellularized samples after the 1st, 2nd, and 3rd cycles (**A**). The biomechanical analysis involved the determination of σΤrans (**B**), εΤrans (**C**), σUTS (**D**), εUTS (**E**), El-E (**F**), and Col-E (**G**). Statistically significant differences were observed only for σΤrans (*p* < 0.001), εUTS (*p* < 0.01), and El-E (*p* < 0.001) between non-decellularized and decellularized samples.

The evaluation of the decellularization process for the production of acellular whole abdominal wall scaffolds involved the uniaxial testing of non-decellularized and decellularized samples (after the first, second, and third cycles; Figure 4). Specifically, the σΤrans for the non-decellularized and decellularized samples after the first, second, and third cycles was 105 ± 15, 184 ± 15, 208 ± 25, and 218 ± 28 kPa, respectively (Figure 4). The εTrans for the non-decellularized and decellularized samples after the first, second, and third cycles was 309 ± 20, 312 ± 47, 312 ± 45, and 313 ± 36, respectively (Figure 4). The σUTS for the non-decellularized and decellularized samples after the first, second, and third cycles was 899 ± 51, 1026 ± 107, 1028 ± 160, and 1035 ± 136 kPa (Figure 4). The εUTS for the non-decellularized and decellularized samples after the first, second, and third cycles was 681 ± 66, 859 ± 98, 860 ± 55, and 916 ± 160, respectively (Figure 4). The El-E for the non-decellularized and decellularized samples after the first, second, and third cycles was 88.2 ± 8, 101 ±16, 113 ± 22, and 115 ± 14 kPa, respectively (Figure 4). The Col-E for the non-decellularized and decellularized samples after the first, second, and third cycles was 2840 ± 352, 3139 ± 375, 3156 ± 377, and 3161 ± 444 kPa, respectively (Figure 4). Statistically significant differences regarding σΤrans (*p <* 0.001), εUTS (*p <* 0.01), and El-E (*p <* 0.001) were observed between non-decellularized and decellularized samples.

### 3.3. Evaluation of Immunogenicity of Decellularized Whole Abdominal Wall Scaffolds

**Figure 5:**
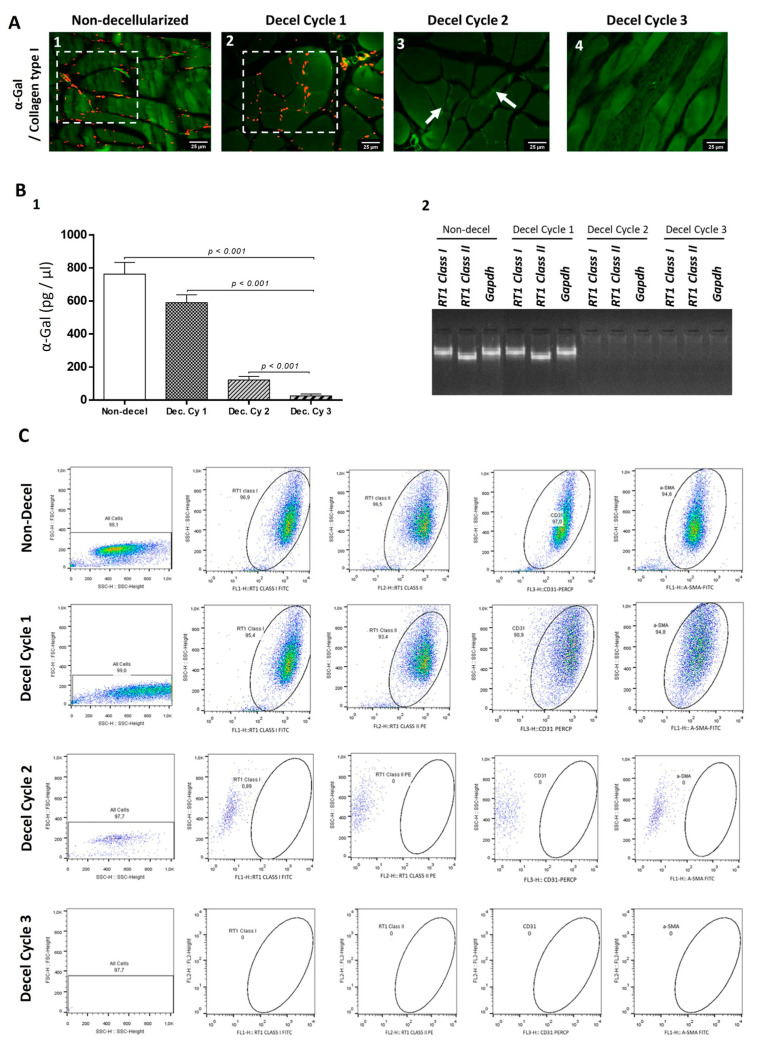
Evaluation of whole abdominal wall immunogenicity before and after the complete decellularization procedure. Indirect immunofluorescence against α-gal epitopes and collagen type I of non-decellularized (**A1**) and decellularized samples after the 1st (**A2**), 2nd (**A3**), and 3rd (**A4**) decellularization cycles. White boxes and arrows indicate the presence of α-gal epitopes in abdominal wall samples. No presence of the α-gal signal was detected in decellularized whole abdominal wall scaffolds after the 3rd cycle. Images presented with the original magnification of 40× and scale bars of 25 μm. Detection of α-gal epitopes in non-decellularized and decellularized abdominal wall samples using the ELISA method (**B1**). A statistically significant difference was observed regarding α-gal expression among all samples (*p <* 0.001). DNA electrophoresis in 1% *v*/*v* agarose gel regarding the expression of *RT1 class I*, *RT1 class II*, and *Gapdh* in non-decellularized and decellularized samples (**B2**). No detection of the PCR products *RT1 class I*, *RT1 class II*, and *Gapdh* was observed in decellularized samples after the 2nd cycle. Quantification of α-gal epitopes in non-decellularized and decellularized samples after the 1st, 2nd, and 3rd decellularization cycles (**B2**). A statistically significant difference regarding α-gal epitopes was found between native and decellularized samples (*p <* 0.001). Flow cytometric analysis of RT1 class I, RT1 class II, CD31, and α-SMA in non-decellularized and decellularized samples (**C**). The RT1 class I, RT1 class II, CD31 and α-SMA were detected only in non-decellurized (expression > 94%) and decellularized samples after the 1st cycle accomplishment (expression > 90%). No detection of the aforementioned proteins were observed in decellularized samples after the 2nd and 3rd cycle.

To further evaluate the immunogenicity of non-decellularized and decellularized abdominal wall scaffolds, the detection of α-gal epitopes using the indirect immunofluorescence approach was performed. α-Gal epitopes were successfully detected in non-decellularized and decellularized samples after the first and second cycles (Figure 5). However, no presence of α-gal epitopes was detected in decellularized samples after the third cycle (Figure 5). Furthermore, the quantification of α-gal epitopes with the ELISA method was performed to further verify the indirect immunofluorescence results. Interestingly, the concentration of α-gal epitopes in non-decellularized samples was 763 ± 67 pg/μL, while in decellularized samples after the first, second, and third cycles, it was 591 ± 45, 122 ± 21, and 25 ± 11 pg/μL, respectively (Figure 5). Statistically significant differences regarding the presence of α-gal epitopes were observed between non-decellularized and decellularized samples (*p* < 0.001).

Moreover, total mRNA was extracted from non-decellularized and decellularized samples and reverse-transcribed, and the cDNA produced was used for the determination of *RT1 class I/II* and *Gapdh*. *RT1 class I/II* and *Gapdh* were only detected in non-decellularized and decellularized samples after the first cycle. No expression of the aforementioned genes was detected in decellularized abdominal wall scaffolds after the second and third cycles (Figure 5). Further verification of these results was achieved using the flow cytometric approach against RT1 class I/II, CD31, and α-SMA. Specifically, the detected expression for the previously mentioned molecules was above 95% for the non-decellularized samples and more than 93% for the decellularized samples after the first cycle. However, the expression of RT1 class I/II, CD31, and α-SMA was below 1% for the decellularized abdominal wall scaffolds after the second and third cycles (Figure 5). 

## 4. Discussion

In the field of reconstructive surgery, the use of appropriate prostheses for the restoration of abdominal wall defects represents one of the greatest challenges of the past decade. For this purpose, the application of a non-immunogenic biological scaffold using state-of-the-art tissue engineering methods for abdominal wall defect restoration may represent a promising alternative source of transplants. This study aimed to produce full-thickness abdominal wall scaffolds using a validated decellularization approach. For this purpose, rat-derived abdominal wall samples were decellularized appropriately to produce acellular non-immunogenic biological scaffolds to serve as potential grafts for future abdominal wall tissue restoration. Histological analysis revealed that complete decellularization of full-thickness abdominal wall scaffolds was achieved after the accomplishment of the third cycle. Cell removal was initiated after the first decellularization cycle; however, complete cell and nuclear remnants were eliminated by the end of the proposed protocol. In addition, decellularized abdominal wall scaffolds (after the third decellularization cycle) were characterized by the preservation of collagen, a significant ECM component. In contrast, sGAGs appeared to be significantly removed after the application of the decellularization protocol. Regarding the elastin content, in addition to its low presence in the abdominal wall, no significant alteration between non-decellularized and decellularized samples was observed. The preservation of collagen in decellularized samples was further confirmed with indirect immunofluorescence (against collagen type I in combination with DAPI). Moreover, collagen type I fibers in decellularized samples retained their alignment both in the circumferential and in the longitudinal axis in the same way as the non-decellularized samples. The preservation of collagen type I in decellularized samples is of significant importance, being one of the major proteins of the ECM structure, thus contributing to a great number of processes, such as hemostasis performance and regulation of the wound-healing process in the injured area [41]. In addition to its function in tissue healing, collagen type I plays a significant role in the recruitment and attachment of specific cellular populations to the wounded region through binding to arginine–glycine–aspartic acid (RGD) binding motifs [34,35,37,38,42,43,44,45,46,47]. Indeed, various cellular populations, such as fibroblasts, pericytes, and mesenchymal stromal cells, can bind to collagen fibers through the interaction of α1β1, α2β1, and ανβ1 with the RGD binding motifs of well-aligned collagen fibers, thus further promoting wound healing and tissue regeneration [34,35,37,38,42,43,44,45,46,47]. Specifically, it has been shown that integrin-mediated binding with the RGD motifs can lead to an important intracellular signaling cascade through the upregulation of ILK–NF-κb and GSK3β–AP1–cyclinD1, promoting in this way cell survival, migration, and proliferation to the injured site [41,42,43,44,45,46,47,48,49,50]. In addition, sGAGs also play an important role in collagen fiber orientation, and their loss after the decellularization process may affect the ECM structure and mechanical properties of the scaffold produced [51,52]. However, the results of this study seem to be in accordance with previously performed works, where also significant differences in the sGAG content were observed in decellularized samples. Importantly, in the studies by Luo et al. [53] and Gui et al. [54], the levels of the sGAG content of decellularized heart valves and umbilical arteries, respectively, were lower compared to the non-decellularized tissues. This can be explained by the knowledge of the anionic nature of SDS, which is used as a basic detergent in all these studies [30]. SDS can interfere with the negatively charged sites of sGAGs and thus can efficiently remove them from the decellularized tissues [30,52].

These results regarding collagen, sGAG, and cell removal were also confirmed with quantification assays performed in this study. Importantly, cell removal was further confirmed by the low levels of the DNA content isolated from decellularized samples. Indeed, the performance of each decellularization cycle appeared to be lower compared to the previous one. Finally, after the third decellularization cycle, the DNA concentration was below 50 ng/μL dry tissue, thus confirming further the criteria for successful tissue decellularization, as outlined by Gilbert et al. [30] and Crapo et al. [55]. In this study, the DNA concentration was determined through the measurement of a 260 nm/280 nm ratio with a spectrophotometer, avoiding in this way false quantification results occurring using commercial kits, such as the Picogreen assay. Most of these commercial kits determine only double-stranded (ds) DNA, without measuring single-stranded (ss) DNA, which may also be present, especially in decellularized tissues [52,56]. Using a spectrophotometer, genetic material quantification is most accurate, representing better the DNA content of the decellularized tissues. It is also widely known that both ds- and ssDNA can initiate a specialized immune response against the implanted graft, which could result in significant adverse reactions to the host [52,56,57]. Biomechanical properties play an important role in tissue mechanics and therefore were evaluated before and after the decellularization process applied to the full-thickness abdominal wall scaffolds. Interestingly, the decellularized abdominal wall scaffolds were characterized by higher values of σ_Trans_, ε_Trans_, σ_UTS_, ε_UTS_, El-E, and Col-E compared to the non-decellularized samples, suggesting the adaptation of a stiffer behavior by the produced scaffolds. Also, differences in biomechanical properties were observed after each decellularization cycle. Overall, the increase in abdominal wall scaffold strength is related to the ECM structure. Considering that collagen fibers are responsible for the preservation of tissue integrity during overload differences in the corresponding anatomical area, changes in collagen orientation play an important role in the altered mechanical properties of the produced scaffolds. In addition, the decellularized abdominal wall scaffolds were characterized by greater extensibility than the non-decellularized samples, which is further related to the disorganized collagen fiber alignment. To assess the relationship between the ECM structure and biomechanical properties, extensive histological analysis of all study samples in both circumferential and longitudinal directions was performed in this study. However, histological analysis results did not reveal any change in the collagen fiber orientation (in both axes). Moreover, the indirect immunofluorescence results against collagen type I confirmed further the results obtained from the histological staining, thus confirming the preservation of collagen fiber alignment after the decellularization procedure. In addition to collagen fibers, elastin fibers also play an important role in tissue mechanical strength and extensibility. However, the elastin content of the abdominal wall is relatively low and elastin fibers are mostly disorganized compared to other tissues, such as elastic vessels (e.g., thoracic or abdominal aorta). However, no change in the elastin content was observed between non-decellularized and decellularized abdominal wall samples. The explanation regarding the stiffer behavior of the decellularized scaffolds may be attributed to the cellular population elimination in the produced scaffolds. Indeed, SMCs and ECs, in addition to their important role in other biological processes, have the ability to retain the orientation of collagen fibers, thus contributing further to the preservation of ECM integrity. Based on the histological analysis, the collagen fibers in the non-decellularized samples were fully crimped, while in the decellularized scaffolds, they became uncrimped after each decellularization cycle. In addition to this phenomenon, the reduction in the sGAG content observed in decellularized scaffolds may also play a role in collagen crimping. It is known that sGAGs form large polymers called proteoglycans, which actively contribute to collagen crosslinking [58]. The significant reduction in sGAGs, in combination with cellular elimination, may lead to an important change in collagen crosslinking, thus affecting further their crimping. Similar alterations in biomechanical properties have also been revealed in the past in other structures, such as the aorta, intestine, and elastic vessels, and have been extensively reported by Sexton et al. [59], Stehbens et al. [60], Sokolis et al. [61], and Stergiopoulos et al. [60]. Specifically, Stergiopoulos et al. [62] indicated in their model that the alteration of the biomechanical properties acquired by decellularized scaffolds cannot be the mere result of cellular elimination only but that further structural ECM changes may provoke altered mechanical behavior. In a similar way, there is substantial evidence for the physical link between SMCs, ECs, and ECM components, which may further contribute to the existence of residual forces in the abdominal wall. However, decellularization causes alterations at both structural and cellular levels of the ECM; thus, prestressed fibers no longer exist, and this may be the explanatory cause of the stiffer behavior of decellularized abdominal wall scaffolds. In addition to this evidence for altered mechanical properties, stiffer abdominal wall scaffolds have been observed in other studies conducted with similar preparation methods. Specifically, Sanchez et al. [36], Buell et al. [37], and Chiu et al. [38] demonstrated decellularized abdominal wall constructs with similar biomechanical properties as those presented herein. The immunogenicity of decellularized scaffolds constitutes another important parameter for them to serve as potential biological grafts. To assess whether the proposed decelluarization protocol can be submitted for the preparation of acellular scaffolds of animal origin, the determination of α-gal epitopes was performed. Complete elimination of α-gal epitopes was achieved after the third decellularization cycle, as indicated by histological and quantification analysis results. α-Gal epitopes have been considered main immunogenic antigens and have been related to hyper-acute rejection when xeno-transplantation was performed in the past [63,64,65]. Currently, there is a great amount of effort to produce α-Gal-epitope-free knockout pigs, which can offer a solution for a global shortage of organs; however, this attempt is costly [66,67]. In the context of biological scaffold production originating from animal models, the decellularization method could potentially assist with this issue. SDS, a detergent of decellularization, is known for its ability to break hydrophobic interactions and ionic and hydrogen bonds, and thus, it can successfully remove negatively charged proteins and genetic material. In this way, SDS has been shown to have the potential to interfere with α-gal epitopes, forcing their elimination by the tissue’s ECM. Although efforts to remove animal-derived antigens from biological scaffolds have been made, α-gal epitopes have been found in transplants, such as heart valves, SIS-ECM, and vessel grafts, impairing the graft longevity after transplantation [63,64,65,66,67,68,69,70]. In this study, the proposed decellularization protocol successfully eliminated α-gal epitopes from the produced scaffolds, thus decreasing their potential immunogenicity upon implantation [68,69,70]. Moreover, molecular and cytometry analysis revealed the absence of both cellular populations, e.g., ECs and SMCs, and other antigenic epitopes, e.g., *RT1 classes I* and *II*. RT1 class I and class II comprise the analog genes of histocompatibility in rats [71,72]. Histocompatibility antigens are actively implicated in transplantation and graft survival, and their removal may be related to the production of universal biological transplants. In a previous study conducted by our lab, the same decellularization protocol was applied in human umbilical arteries, where also the elimination of HLA class I and II antigens was confirmed after the accomplishment of a comprehensive shotgun quantitative proteomic analysis [73]. These results suggest further that the proposed decellularization protocol successfully produces low-immunogenic full-thickness abdominal wall scaffolds derived from Wistar rats. The study presented herein demonstrates a proof-of-concept protocol for the production of acellular full-thickness abdominal wall scaffolds for potential use as transplants. In this study, an already established decellularization protocol, which has been validated by our research team in several tissues and organs, including the human umbilical cord artery, Wharton’s jelly tissue, the esophagus, and kidneys, was evaluated for the proposed tissue [40,50,73,74,75,76]. In the past, other research groups have also tried to produce full-thickness abdominal wall scaffolds, with contradictory results. Specifically, Chiu et al. [38] demonstrated the production of the porcine acellular dermal matrix (ADM) decellularized with the use of supercritical carbon dioxide (SCCO_2_). Chiu et al. [38] successfully produced the ADM with this method; however, both the ECM ultrastructure and the main components appeared to be significantly affected by SCCO_2_. There was a significant difference between non-decellularized and decellularized samples regarding the histological stain intensity. In addition, ADM samples under higher magnification were characterized by alterations in ECM integrity. In contrast, in our study, no alteration in histological stain intensity between non-decellularized and decellularized samples (from the first, second, or third cycle) was observed. Only the sGAG content was affected by the proposed decellularization protocol, as confirmed by both histological and quantification results. However, in the majority of the studies focused on the production of decellularized scaffolds, a similar loss of sGAGs has been observed [40,53,54,73,74,75,76]. Moreover, sGAG content loss and cellular population elimination are responsible for the altered mechanical properties of acellular scaffolds. In addition, although the studies conducted by Sanchez et al. [36] and Buel et al. [37] have indicated the production of acellular abdominal wall scaffolds, the tissue ECM was affected in a similar way as in the study by Chiu et al. [38]. Taking into account the available data from the literature, in our study, a comprehensive evaluation of the decellularized scaffold histology (circumferential vs. longitudinal axis) was performed in order to reach safe conclusions.

In conclusion, the results of this study clearly showed the successful production of a full-thickness rat-derived abdominal wall scaffold. Moreover, major immunogenic epitopes, such as α-gal and RT1 class I and II antigens, were removed; thus, the produced scaffolds were characterized by low immunogenicity. Considering our results, in the near future, a second study will be primarily prepared, where implantation of the produced full-thickness decellularized abdominal wall scaffold will be performed in an animal model in order to investigate deeper the behavior of the transplant in terms of mechanical strength, capability for tissue integration, non-immunogenic nature, and also underlying tissue remodeling mechanisms. The proposed decellularization protocol could be assessed for the production of acellular abdominal wall scaffolds derived from larger animal models (porcine origin) or human cadaveric donors. Furthermore, this study exploited the possibility of the production of non-immunogenic acellular meshes, with good tissue integrity and mechanical properties, that potentially could be used as transplants in abdominal wall defects. Such acellular biologic scaffolds, with in vivo tissue remodeling properties, may be considered superior to the current gold-standard methods (e.g., synthetic scaffolds). Hence, a comprehensive evaluation of the properties of decellularized biologic scaffolds is required to be performed in the future to ensure appropriate FDA clearance. Moreover, specific regulatory concerns regarding the use of decellularized abdominal wall scaffolds, especially those derived from cadavers, should also be considered before their commercialization [32]. Under these circumstances, the ultimate goal will be the use of these scaffolds by clinicians in the near future, bringing them one step closer to their clinical utility.

## Figures and Tables

**Table 1:** Primer pairs for the PCRs.

Gene	Gene ID	Forward Primer	Reverse Primer	Amplicon Size
*RT1 Class I*	309627	CAGATCCCCCAAAGGCACAT	CAGATCCCCCAAAGGCACAT	253
*RT1 Class II*	309622	CTTCCTTACCCGGGTGGAAC	TCTGATCACGAGCCGTATGC	316
*Gapdh*	24383	GGCCCGGAGTCTAAAGTAGC	GGCGGCCAGTTACCATAAGT	234

## Data Availability

The data that support the findings of this study are available upon request from the corresponding author, P.M. The data are not publicly available due to information that could compromise the privacy of patients.

## Supplementary Materials

The following supporting information can be downloaded at https://www.mdpi.com/article/10.3390/bioengineering10080913/s1: Table S1. DNA quantification and biochemical analysis, including determination of the hydroxyproline and sGAG content of non-decellularized and decellularized samples.
