# Supplementary material for: Efficient Decellularization of the Full-Thickness Rat-Derived Abdominal Wall to Produce Acellular Biologic Scaffolds for Tissue Reconstruction: Promising Evidence Acquired from In Vitro Results
Source: Bioengineering (Basel). 2023 Aug 1;10(8):913. doi: 10.3390/bioengineering10080913 (PMC10451677; doi:10.3390/bioengineering10080913)
Supplement: Supplementary file 1 [file bioengineering-10-00913-s001.zip › bioengineering-2517889-supplementary.pdf]

Supplementary Materials

Table S1. DNA quantification and biochemical analysis including the determination of the hydroxyproline and sGAG content of non-decellularized and decellularized samples. Statistically significant differences regarding the DNA content ($p < 0.001$), hydroxyproline content ($p = 0.021$) and sGAGs ($p < 0.001$) were observed between non-decellularized and decellularized samples.

	**Non-decel**	**Decel Cycle 1**	**Decel Cycle 2**	**Decel Cycle 3**	***p value***
DNA (ng /mg dry tissue)	1363 ± 87	542 ± 73	167 ± 35	48 ± 12	<0.001
Hydroxyproline (µg / mg dry tissue)	26 ± 5	22 ± 4	20 ± 3	20 ± 2	0.021
sGAGs (µg / mg dry tissue)	9.1 ± 1.7	5.3 ± 1.7	2.1 ± 0.6	1.1 ± 0.3	<0.001
